# Pandora's Box

**DOI:** 10.1192/bji.2020.61

**Published:** 2021-02

## Happy gut for good sleep

It is now well established that our gut microorganisms have a major role in our health. Keeping them active and in good condition is to the benefit of our brain function and mental health.

In a simple experiment in mice, Japanese researchers examined the relationship between gut microbes and sleep. They gave one group of mice a powerful cocktail of antibiotics that knocked out their gut bacteria, and compared the intestinal contents of these mice with a group of mice who had the same diet but had not been exposed to antibiotics. They found a major difference between the gut metabolite concentrations of the two groups. On further examination, they observed that the metabolites most affected by the antibiotics were those that were normally involved in the production of neurotransmitters; the tryptophan–serotonin pathway was most affected. The antibiotic-treated mice with microbiota depletion had higher tryptophan concentrations than the controls, but serotonin concentrations were almost non-existent, suggesting that in the absence of the gut microbes, tryptophan could not be converted to serotonin. Vitamin B6, which accelerates the production of serotonin as well as that of dopamine, was also deficient in the antibiotic-treated group.

Sleep analysis based on electroencephalography and electromyography showed that the mice in the experimental group spent less time in non-rapid eye movement (NREM) sleep during the light phase and more in NREM and rapid eye movement (REM) sleep during the dark phase, while REM sleep episodes were increased in both light and dark phases. Their conclusion was that the state of the intestinal microbiota is important to the sleep/wake architecture and that this occurs via changes in the concentrations of relevant neurotransmitters.

Ogawa Y, Miyoshi C, Obana N, Yajima K, Hotta-Hirashima N, Ikkyu A, . Gut microbiota depletion by chronic antibiotic treatment alters the sleep/wake architecture and sleep EEG power spectra in mice. Sci Rep 2020; 10(1): 19554.3317759910.1038/s41598-020-76562-9PMC7659342

## A cocoa a day keeps your brain in shape

Are you one of those people who turn to chocolate to boost their brain serotonin when feeling down in mood? Well, it might be a good idea to do so also when you need to task your brain. Previous studies found that flavanols contained in cocoa, as well as in other plants including berries, apples, and tea, protect from vascular disease but also from cognitive ageing.

A recent study explored these findings further using a randomised double-blind, within-subject approach in healthy young volunteers. They examined cerebrovascular reactivity using a CO_2_-breathing challenge to induce hypercapnia before and after either low or high intake of cocoa flavanols, and measured cortical haemoglobin concentrations using functional near-infrared spectroscopy to assess the amplitude and time course of brain oxygenation responses. They found that cocoa flavanols made brain oxygenation responses to induced hypercapnia both faster and greater. In addition, the participants showed better performance on cognitive tasks of escalating levels of difficulty when the cognitive demand was high. Further analysis showed that those who showed good oxygenation responses to hypercapnia also showed better responses to the higher cognitive challenge. They concluded that similar vascular mechanisms underlie both the peripheral and brain effects of flavanols, probably operating via the nitrous oxide pathway.

Gratton G, Weaver SR, Burley CV, Low KA, Maclin EL, Johns PW, . Dietary flavanols improve cerebral cortical oxygenation and cognition in healthy adults. Sci Rep 2020; 10: 19409.3323521910.1038/s41598-020-76160-9PMC7687895

***Covid-19 has turned our lives upside down, and Pandora, having managed to keep this page virus-free so far, can do so no more. There are some new research findings worth thinking about.***

## Is the corona virus getting up your nose?

Have you wondered why those infected by the corona virus lose their sense of smell and taste? Initially, it was thought that the virus had a predilection for the respiratory system, but we now know that it affects other essential body systems, including the central nervous system – but how does the virus get there?

Researchers at Charité – Universitatsmedizin Berlin have recently identified the route by which it finds its way into the brain. Using a variety of methods, they examined post-mortem brain tissue from people who had died from Covid-19, and demonstrated the presence of SARS-CoV-2 RNA and protein in anatomically distinct regions of the nasopharynx and brain. They found that the virus could transcend the neural–mucosa interface and penetrate neuroanatomical structures, including the respiratory and cardiovascular control centres in the medulla oblongata. This is an interesting finding that may significantly contribute to the very serious respiratory and cardiovascular difficulties that Covid-19 patients experience.

SARS-CoV-2 appears to move along nerve cells, crossing over from one to another, although the authors think it is likely that the virus is also transported via the blood circulation, as it was found in the walls of blood vessels. The study also found evidence of immune cell activation in the brain, as well as brain tissue damage caused by thromboembolism.

Meinhardt J, Radke J, Dittmayer C, Franz J, Thomas C, Mothes R, . Olfactory transmucosal SARS-CoV-2 invasion as a port of central nervous system entry in individuals with COVID-19. Nat Neurosci 2020. Available from: 10.1038/s41593-020-00758-5.33257876

## A friendly pet or a cuddly robot?

As humans, we are highly socialised creatures and isolation is bad for our health. Nevertheless, many people have to cope with loneliness, and this has been part of life for many more since the Covid pandemic. The pandemic has brought misery to people around the world, killing a million and a half loved ones and depriving many of direct human contact, with resulting harm to their mental health and well-being. Many turned to their pets for companionship and cuddles. It has been observed that since the pandemic-imposed lockdown and social distancing, animal shelters and breeders have been inundated with requests for pets.

Australian researchers have just published a paper stressing the invaluable role pets can have in such difficult times and the importance of physical touch, a sense they claim has been taken for granted and overlooked. They interviewed 32 people, 90% of whom said that touching their pets comforted and relaxed them. Many reported their animals sensing them feeling unwell and responding by getting physically close to them. The authors argue that not enough attention has been paid to the physical and mental health benefits derived from the presence of pets, particularly for people with limited or no access to human touch. They recommend that hospitals, hospices and old age caring facilities encourage pet connections with residents, particularly in these times of Covid pandemic-imposed isolation.

What if, for a variety of reasons, you do not have access to human or pet contact? Are there other options? Would you consider a cuddly robot?

Researchers at Ben Gurion University examined whether a furry social robot could have a beneficial effect. They used PARO, a Japanese robot that emits seal-like sounds and moves its head and flippers when spoken to or touched, and studied its effects on young adults. Sixty-three participants who were exposed to PARO were compared with 20 controls, who had no contact with PARO at any time. The researchers measured perceived pain, happiness state and salivary oxytocin levels in both groups. In the PARO group, pain was assessed under three conditions: before access to the cuddly robot, in its presence but with no touching of the robot, and touching the robot. The participants interacted with the robot for 1 h. The results showed a reduction in pain ratings and oxytocin levels and an increase in happiness ratings in the PARO group compared with the baseline. The touch condition was associated with a more marked decrease in pain compared with no touch. Those with higher perceived ability to communicate with PARO experienced a more marked reduction in pain, which correlated with their positive perceptions.

Young J, Pritchard R, Nottle R, Banwell H. Pets, touch, and COVID-19: health benefits from non-human touch through times of stress. J Behav Econ Policy 2020; 4 (COVID-19 Special Issue 2), 25–33.

Geva N, Uzefovsky F, Levy-Tzedek S. Touching the social robot PARO reduces pain perception and salivary oxytocin levels. Sci Rep 2020; 10: 9814.3255543210.1038/s41598-020-66982-yPMC7299999

## Hedonism now or long-term goals

The Covid pandemic has affected us in many ways. In addition to searching for comfort, many of us set ourselves long-term goals such as maintaining fitness through exercise, healthy eating, learning a new language or skill and many other goals. Research confirms that such pursuits, which require self-control, are associated with good outcomes. The truth is, however, that many of us pursue more hedonistic goals such as relaxing on the sofa, although we may feel guilty about it. For many of us, having intrusive thoughts of activities we should be doing instead spoils the pleasure of lazing about.

The authors of a recent publication put our minds at ease on the subject. They carried out a series of studies in which they developed a questionnaire to measure people's trait hedonic capacity and proceeded to examine whether this was related to well-being. They also assessed the occurrence of intrusive thoughts. They found that people's trait hedonic capacity was related positively to a sense of well-being. Further examination confirmed that the presence of intrusive thoughts had a major adverse effect on hedonic success. In their final two studies, they demonstrated that trait hedonic capacity predicts successful hedonic goal pursuit in everyday life.

They happily conclude that ‘the pursuit of hedonic and long-term goals' needn't be in conflict with one another. They are both important and can complement each other in achieving well-being and good health. Just find the right balance!

Bernecker K, Becker D. Beyond self control: mechanisms of hedonic goal pursuit and its relevance for well being. J Pers Soc Psychol 2020; 78: 53–63.10.1177/014616722094199832715943

